# Intracellular calcium levels as screening tool for nanoparticle toxicity

**DOI:** 10.1002/jat.3160

**Published:** 2015-05-14

**Authors:** Claudia Meindl, Tatjana Kueznik, Martina Bösch, Eva Roblegg, Eleonore Fröhlich

**Affiliations:** a Center for Medical Research, Medical University of GrazAustria; b Institute of Pharmaceutical Sciences, Department of Pharmaceutical Technology, Karl-Franzens-University of GrazAustria

**Keywords:** Nanoparticles, cytotoxicity, apoptosis, lysosomes, calcium imaging

## Abstract

The use of engineered nano-sized materials led to revolutionary developments in many industrial applications and in the medical field. These materials, however, also may cause cytotoxicity. In addition to size, surface properties and shape were identified as relevant parameters for cell damage. Cell damage may occur as disruption of membrane integrity, induction of apoptosis and by organelle damage. Generation of oxidative stress may serve as an indicator for cytotoxicity. Effects occurring upon short contact of particles with cells, for instance in the systemic blood circulation, could be identified according to increases of intracellular [Ca^2+^] levels, which are caused by variety of toxic stimuli. Negatively charged, neutral and positively charged polystyrene particles of different sizes were used to study the role of size and surface properties on viability, membrane disruption, apoptosis, lysosome function, intracellular [Ca^2+^] levels and generation of oxidative stress. Silica particles served to test this hypothesis. Twenty nm polystyrene particles as well as 12 nm and 40 nm silica particles caused membrane damage and apoptosis with no preference of the surface charge. Only 20 nm plain and amine functionalized polystyrene particles cause oxidative stress and only the plain particles lysosomal damage. A potential role of surface charge was identified for 200 nm polystyrene particles, where only the amidine particles caused lysosomal damage. Increases in intracellular [Ca^2+^] levels and cytotoxicity after 24 h was often linked but determination of intracellular [Ca^2+^] levels could serve to characterize further the type of membrane damage. © 2015 The Authors. *Journal of Applied Toxicology* Published by John Wiley & Sons Ltd.

Nano-sized materials may cause cytotoxicity. Negatively charged, neutral and positively charged polystyrene particles of different sizes and silica nanoparticles were used to study the role of size and surface properties on viability, membrane disruption, apoptosis, lysosome function, intracellular [Ca^2+^] levels and generation of oxidative stress. Small polystyrene particles and silica particles caused membrane damage and apoptosis with no preference of the surface charge. Increases in intracellular [Ca^2+^] levels could be used as a screening tool for cytotoxicity.

## Introduction

Nanoparticles (NPs), which are contained in a variety of products of daily use and in medical products, may cause cytotoxicity. Size, shape, surface charge and surface hydrophobicity, and heavy metal content determine cytotoxicity. Lack of biodegradability and generation of reactive oxygen species (ROS) and nitrogen species are indicators for toxic action (Fröhlich, 2013). With few exceptions, smaller NPs are more toxic than larger ones (Shang *et al*., 2014). It is also known that fiber-shaped nanomaterials, such as carbon nanotubes, react more toxically than spherical particles, such as fullerenes (Poland *et al*., 2008; Takagi *et al*., 2008). Surface properties, such as charge and hydrophobicity are additional determining factors for cytotoxicity. Cationic NPs not only permeated cell layers to a higher degree (Roblegg *et al*., 2012), they also lead to the disruption of plasma membrane integrity by causing holes or increasing the size of pre-existing holes in the plasma membrane (Lin *et al*., 2010, 2011; Mecke *et al*., 2004). NPs with hydrophobic surfaces, e.g., oleic acid coated nickel ferrite and stearic acid coated titanium oxide (TiO_2_) show higher cytotoxicity than the respective non-coated particles (Onuma *et al*., 2009; Teubl *et al*., 2015; Yin *et al*., 2005).

The most common mode of cytotoxic actions of non-biodegradable NPs is the generation of ROS, which may cause organelle damage and may lead to apoptosis (Manke *et al*., 2013; Nel *et al*., 2009). Of all the intracellular organelles NPs have the most intense contact with lysosomes as most active uptake routes occur via endocytotic routes that deliver their content to lysosomes (Kou *et al*., 2013). Other organelles, such as nuclei and mitochondria, restrict the access of NPs by membranes (Panariti *et al*., 2012) and NPs reach these organelles by damage of the surrounding membranes. Membrane damage can occur by ROS and lysosomal membranes are particularly sensitive to ROS (Olsson *et al*., 1989). On the other hand, lysosomes may increase intracellular ROS levels by release of toxic ions (Sabella *et al*., 2014). Alternatively, lysosomes may increase ROS production by damaging mitochondria by releasing lysosomal enzymes. The main role of lysosomes is degradation of the intracellular molecules and organelles (autophagy) but they are also involved in the regulation of intracellular [Ca^2+^] levels. Lysosomes contain high concentrations of Ca^2+^ and lysosome dysfunction results in aberrant regulation of intracellular [Ca^2+^] levels (Lopez-Lorente *et al*., 2013). The decreased capacity for Ca^2+^ uptake in damaged lysosomes leads to increased intracellular [Ca^2+^] levels. Lastly, oxidative stress is also linked to high intracellular [Ca^2+^] levels (Ermak & Davies, 2002).

TiO_2_, Ag and ZnO NPs and quantum dots, have been reported to increase intracellular [Ca^2+^] levels (Huang *et al*., 2009) and release of Ca^2+^ from intracellular stores and entry through membrane channels (Tang *et al*., 2008; Viswaprakash *et al*., 2009; Zhao *et al*., 2009). Changes in intracellular [Ca^2+^] levels mediate a variety of cellular processes. High [Ca^2+^] levels mediate plasma membrane repair but may also induce cell death (Sharei *et al*., 2014; Verkhratsky, 2007). Changes in intracellular [Ca^2+^] levels, however, may also precede membrane damage (Edelstein, 2000).

**Figure 1:**
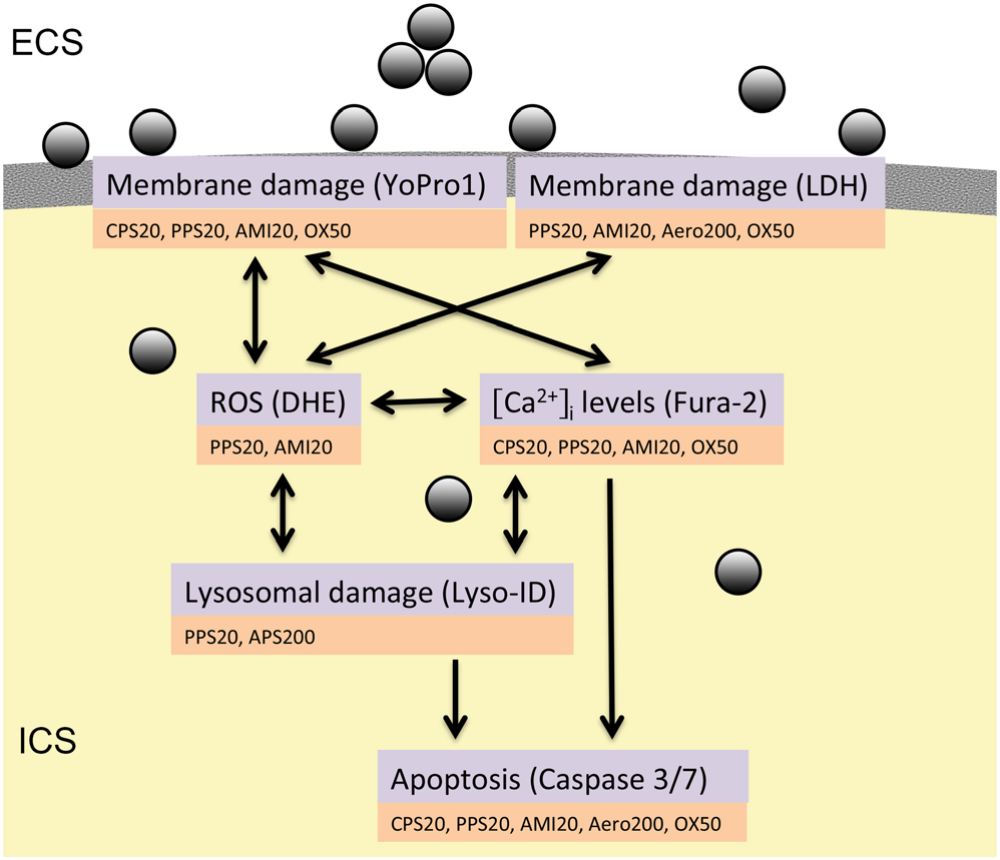
NPs can cause disruption of membrane integrity causing small or large holes. They can generate ROS, cause lysosome damage, increase intracellular [Ca^2+^] levels and induce apoptosis. Assays that were employed to study these effects and particles with significant action in these assays are indicated. Only effects recorded in concentrations up to 100 µg ml^−1^ for 24 h and up to 750 µg ml^−1^ for 30 s as used in Ca^2+^ imaging are mentioned. DHE, dihydroethidium; ECS, Extracellular space; ICS, intracellular space; LDH, lactic dehydrogenase; ROS, reactive oxygen species.

One aim of the study was to identify a link between different surface properties and cytotoxicity. Known cytotoxic targets of NP action, namely disruption of membrane integrity, induction of apoptosis, lysosomal damage and generation of ROS, were used as read-out. Another aim was to find out whether increases in intracellular [Ca^2+^] levels could be used to identify cytotoxic NPs. NPs present in the systemic blood circulation may have only short contact times with endothelial cells and blood cells. Routine cytotoxicity testing assesses effects after 24 h and NPs could exhibit another extent of membrane toxicity after short contact times. Owing to the short contact time with cells, measurements of intracellular [Ca^2+^] levels may be useful to classify the severity of plasma membrane damage. As illustrated in the overview (Fig.1) read-out parameters used in this study are interconnected. In this figure, particles that caused significant effects in concentrations up to 100 µg ml^−1^, for 24 h in the employed assays, are mentioned because higher concentrations under this condition appear unrealistic *in vivo* (Strobel *et al*., 2014). Owing to the technology of the measurement, changes in intracellular [Ca^2+^] levels are determined after a contact time with the cells of only 2 min and, for this detection, also effects at higher than 100 µg ml^−1^ are included.

Polystyrene (PS) particles were used as model particles because they do not release metal ions and lack ROS production in an abiotic system (Xia *et al*., 2008). To find out whether intracellular [Ca^2+^] levels could also identify cytotoxic NPs made from other materials fumed silica particles (Aerosil®) of two different sizes, 12 nm Aerosil200 and 40 nm Aerosil®OX50 particles, with different cytotoxicity were also tested. Aerosil® fumed silica NPs are used in a variety of food applications, such anti-caking agent in spices, seasonings, egg or milk powder, and cappuccino https://www.aerosil.com/lpa-productfinder/page/productsbytext/detail.html?pid=1855&lang=en. Personal care products, such as toothpaste, sun protection, creams and lotions, as well as gels and creams, semi-solid and solid dosage forms in pharmaceuticals contain Aerosil® 200.

For the cytotoxicity testing, EAhy926 endothelial cells were used. Owing to the higher sensitivity changes in intracellular [Ca^2+^], levels were determined in SH-SY5Y cells.

## Materials and Methods

Carboxyl PS (CPS) latex beads (20 and 200 nm; Invitrogen, Vienna, Austria), plain PS particles (PPS) (20 nm and 200 nm; ThermoScientific, Braunschweig, Germany), amine PS particles (AMI) 20 nm (Merck Chemicals and Life SCiences, Vienna, Austria) and 200 nm (Invitrogen), and amidine PS particles (APS) (20 nm and 200 nm, Invitrogen) were used. Aerosil®200 (Aero200; 12 nm) and Aerosil®OX50 (OX50; 40 nm) were obtained from Degussa, Frankfurt, Germany.

The human endothelial cell line EAhy926 (kind gift from Dr. C. J. Edgell) was cultured in Dulbecco minimal Eagle’s medium (DMEM), 10% fetal bovine serum (FBS), 2 mM L-glutamine and 1% penicillin/streptomycin. Human neuroblastoma cells SH-SY5Y (American Tissue and Cell Culture Collection, Manassas, VA, USA), used for the detection of intracellular [Ca^2+^] changes, were cultured in 90% DMEM/Ham’s F12, 10% FBS, 2 mM L-glutamine and 1% penicillin/streptomycin. All cells were cultured at 37 °C in a humid 95% air/5% CO_2_ atmosphere.

### Characterization of Particles

Physicochemical characterization of the particles was performed by dynamic light scattering using a (Malvern Instruments, Malvern, UK) 3000 HS. For the measurements, particles were diluted with DMEM to concentrations of 200 µg ml^−1^ and sonicated for 20 min. Similar dilutions were performed for testing in Hank’s balanced salt solution (HBSS) and in distilled water. After equilibration of the sample solution at 25 °C, the size and zeta potential were measured at 633 nm and a detection angle of 90° respectively. NNLS software was used for data analysis.

Hydrophobicity was determined by binding of Rose Bengal according to the protocol established by Müller (1991). One hundred µg ml^−1^ PS particles were incubated for 3h at room temperature with 10, 20, 30 and 40 µg ml^−1^ Rose Bengal (Sigma-Aldrich, Vienna, Austria), centrifuged for 3 h at 16 000 g and absorbance of the supernatant determined at 544 nm using a plate reader (SPECTRA MAX plus 384; Molecular Devices, Wlaz-Siezenheim, Austria). Control samples were used in each experiment to omit the effect of binding of Rose Bengal to the centrifuge tubes and pipette tip. The binding constant of Rose Bengal was calculated using a Scatchard plot (amount of Rose Bengal_bound_/mg particle vs. free Rose Bengal concentration) according to the equation:

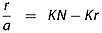


with *r*, amount of Rose Bengal adsorbed (µg µm^−2^); *a*, equilibrium concentration of Rose Bengal (µg ml^−1^); K, binding constant (ml µg^−1^); and *N*, maximum amount bound (µg µm^−2^).

### Formazan Bioreduction by MTS

Cells were exposed to 0, 25, 50, 100, 200 and 400 µg ml^−1^ particles. CellTiter 96® AQueous Non-Radioactive Cell Proliferation Assay (Promega, Mannheim, Germany) was used according to the manufacturer’s instructions. In short, 20 µl of the combined MTS/phenazine methosulfate solution was added to 100 µl of each well. Plates were incubated for 2 h at 37 °C in the cell incubator. Absorbance was read at 490 nm on a plate reader (SPECTRA MAX plus 384; Molecular Devices).

### Lactate Dehydrogenase Release

Cells were exposed to 0, 25, 50, 100, 200 and 400 µg ml^−1^ particles. The CytoTox-ONE™ The Homogeneous Membrane Integrity Assay (Promega) was used according to the manufacturer’s instructions and fluorescence was recorded with an excitation wavelength of 544 nm and an emission wavelength of 590 nm. After subtraction of the blank value, the average fluorescence from the samples was normalized to the maximum lactate dehydrogenase (LDH) release (lysis control).

### Lysosome Function

For the detection of lysosome function, two different pH sensitive dyes (Acridine orange and Lyso ID® Red Dye) were used. Acridine Orange is a lysosomotropic weak base vital dye that stains lysosomes in red (metachromatic staining) and nucleic acids and mitochondria in green (orthochromatic staining). It is used as a functional assay for lysosomes (Del Bino *et al*., 1991). Lyso ID® Red Dye is a cell-permeable small organic probe molecule that spontaneously localized to live acidic organelles. Chloroquine, a lysosomotropic agent known to increase intralysosomal pH (MacGregor *et al*., 1979), was used as the positive control.

*Acridine orange.* After exposure for 24 h to 0, 5, 10, 20 and 40 µg ml^−1^ PS, to negative controls (DMEM) and to positive controls (25 µM chloroquine; Sigma-Aldrich) cells were washed and loaded with 0.5 µM Acridine Orange (Sigma-Aldrich) in phosphate-buffered saline for 30 min at 37 °C. After rinse in phosphate-buffered saline, cells were first evaluated at a FLUOstar Optima (BMG Labtech, Ortenberg, Germany) with 485 nm/520 nm for the green and 584 nm/612 nm for the red channel and viewed at a confocal laser scanning microscope, Vienna, Austria Meta (Zeiss). Images were acquired at 488 nm excitation wavelength using a BP 505–530 nm band-pass detection filter for the green signal and 543 nm excitation wavelength in conjunction with LP 560 for the red signal. Intensity in the different channels was analyzed using Image J software and the ratio of the signal in the red to the green channel per cell was determined. The ratio of the red to the green channel of medium exposed cells was set as 100%.

*Lyso-ID® Red.* Cells were grown in chamber slides and exposed to non-cytotoxic concentrations of PS (0, 5, 10, 20, 40 µg ml^−1^) for 24h. Lyso-ID® Red Detection Kit (Enzo Life Sciences, Lausen, Switzerland) was used according to the protocol provided by the producer. Ten µM chloroquine was used as the positive control. After staining of cells with dye for 20 min in the dark, cells were rinsed in Assay Buffer and covered with a coverslip. The slides were scanned in the TissueFAXS (Tissuegnostics, Vienna, Austria) and analyzed by TissueQuest software. Medium-treated cells were used for the gating.

### Caspase 3/7 Activation

Caspase 3/7 activation was evaluated in cells after 8 h and 24 h to 0, 25, 50, 100, 200 and 400 µg ml^−1^ particles. The Caspase-Glo 3/7 Assay (Promega) was performed according to the instruction manual and luminescence read on a Lumistar (BMG Labortechnik).

### YoPro1/propidium Iodide Staining

Cells were treated for 24 h with 0, 12.5, 25, 50 and 100 µg ml^−1^ particles. The Vybrant Apoptosis Assay Kit no. 4 (Invitrogen) composed of 100 µM YoPro1 and 1.5 mM propidium iodide (PI) was used according to instructions in the manual; 0.5 µl of each dye was added to 1 ml DMEM, gently mixed and incubated for 30 min at 4 °C in the dark. Fluorescence was read at an excitation/emission wavelength of 485/520 nm for YoPro1 and 544/612 nm for PI using a fluorescence plate reader (FLUOstar Optima; BMG Labortechnik) and cells were viewed with a LSM510 Meta (Zeiss) with the following settings: 488 nm/BP 505–550 nm for the green channel (YoPro1) and 543 nm/LP 560 for the red channel (PI). The dose-dependent changes in YoPro1 and PI signals upon incubation with 50, 100 and 200 µM H_2_O_2_ served as positive control.

### Evaluation of Oxidative Stress by Oxidation of Dihydroethidium

Cells were grown for 24 h in cell culture plates and afterwards cultured in the presence of 10 µM dihydroethidium (DHE; Invitrogen) and medium, particles and 100 µM as positive control. Particle concentrations of 0, 12.5, 25, 50, 100, 200 and 300 µg ml^−1^ were tested. Fluorescence was read with 544 nm excitation and 612 nm emission at a fluorometer.

### Calcium Imaging

All images were obtained with A-Plan 20×/0.45 Ph2 objective on an inverted microscope (Axiovert M200; Zeiss). Excitation at 340 and 380 nm was performed with a high-speed polychromator system. All devices were controlled by VisiView software (Visitron systems GmbH, Puchheim, Germany).

Ca^2+^ measurements were performed at ∼37 °C in a heated chamber (QE-1; Warner Instruments Hamden, CT, USA) with a ISM930 pump (Ismatec, IDEX Health & Science GmbH, Wertheim, Germany) and controlled by valve controller VC8 (Warner Instruments) and Micro-Manipulator controller Fast Step Perfusions (Warner Instruments). A bright-field image of a single field of view was captured and used to select regions of interest. Typically, > 100 neurons were imaged for each experiment. The fluorescence emission was monitored at 510 nm for both 380 and 340 nm excitation. The fluorescence emissions (500–530 nm) were monitored by a CCD camera (Roper CoolSnap HQ, Photometrics, Tucson, AZ, USA). An image was captured at each excitation wavelength, and the ratio of fluorescence intensities at excitation wavelengths of 340 and 380 nm was acquired once every 1 or 2 s to monitor the relative changes in Ca^2+^ concentration in each cell as a function of time. Ratiometric analysis was analyzed with computer software (MetaFluor; Molecular Devices).

SH-SY5Y cells (9 × 10^4^) were seeded per poly-l-lysine coated coverslip supplied with a flexiPERM® disc (Sigma-Aldrich, Vienna, Austria). Cells were loaded with 100 µl 8 µM Fura-2 (Life Technologies) in HBSS (0.137 M NaCl, 5.4 M NaCl, 0.25 mM Na_2_HPO_4_, 0.44 mM KH_2_PO_4_, 2 mM NaHCO_3,_ 1.3 mM CaCl_2_, 1.0 mM MgSO_4,_ 5.6 mM D-glucose, pH 7.4) + 2% bovine serum albumin for 30 min at room temperature in the dark, rinsed twice with 100 µl HBSS and incubated with HBSS for a further 30 min in the dark. Particles were suspended in HBSS in concentrations of 0, 12.5, 25, 50, 75, 125, 250, 500 and 750 µg ml^−1^ and treated with ultrasound for 20 min. Experiments were performed in the following sequence: perfusion bathflow; 30 s PS suspension perfusion; 30 s stop (to allow contact of NPs with the cells); and 2 min recording in perfusion. At the end of the recording Ca^2+^ stores were depleted using the ionophore ionomycin (1 µM, Life Technologies) for 1 min. For statistical analysis the fluorescence ratio at excitation 340/380 at time points 120, 150 and 180 s were compared to non-stimulated cultures.

### Statistics

Data are represented as means ± SD from three to four experiments. Data were analyzed with one-way analysis of variance followed by Tukey-HSD *post hoc* test for multiple comparisons (SPSS 19 software). Results with *P* < 0.05 were considered statistically significant.

## Results

Before the cellular assays, size and surface charge were determined in different suspension media. Characterization in deionized water was used to get an indication on the surface charge, which is highly influenced by electrolytes. In buffered solutions, the zeta potential gives only indirect information on the surface charge (Kim & Lawler, 2005). In addition, characterization in HBSS used for the measurements of intracellular [Ca^2+^] levels and in cell culture medium (DMEM without FBS) used for the remaining assays was performed.

APS particles displayed a positive charge in water, while AMI, PPS and CPS particles were negatively charged (Table1). Hydrodynamic sizes of all particles were much larger in HBSS than in water and DMEM. With the exception of APS20 particles, PS particles with a nominal diameter of 20 nm measured < 50 nm in cell culture medium and about 800 nm in HBSS. When suspended in water, cell culture medium and HBSS all particles with the exception of APS20 particles had a negative zeta potential.

**Table 1:** Overview of size and zeta potential in different suspension media. Sizes of the predominant peak are indicated

Particles	Nominal size (nm)	Water		DMEM		HBSS	
		Size (nm)	ζ-Potential (mV)	Size (nm)	ζ-Potential (mV)	Size (nm)	ζ-Potential (mV)
CPS20	20	57	−50	42	−41	740	−20
CPS200	200	275	−29	230	−33	1780	−13
PPS20	20	24	−17	29	−13	763	−13
PPS200	200	262	−49	211	−11	1925	−10
AMI20	20	20	−40	25	−29	823	−24
AMI200	200	224	−37	1415	−9	1327	−12
APS20	20	274	40	717	22	agglom	4
APS200	200	345	55	2831	10	agglom	−1
Aero200	12	261	−17	206	−27	237	−3
OX50	40	546	−20	230	−32	1714	−2

agglom, only agglomerates; DMEM, Dulbecco minimal Eagle’s medium; HBSS, Hank’s balanced salt solution.

All particles were classified as very hydrophilic according to Rose Bengal binding. Aero200 and all 20 nm NPs, except CPS20, showed K values of < 0.02 ml mg^−1^. Two hundred nm NPs, OX50 and both CPS particles showed slightly higher K values between 0.02 and 0.04 ml mg^−1^ Rose Bengal binding. The slightly higher binding of Rose Bengal does not indicate hydrophobic surface properties; non-stabilized (hydrophobic) solid lipid nanoparticles showed binding constants of 4.25 ml mg^−1^ while PEG40-stabilized (hydrophilic) solid nanoparticles had binding constants of 0.036 ml mg^−1^ (Varshosaz *et al*., 2012). Binding constants of about 0.04 ml mg^−1^ were indicated as the lower limit for hydrophobicity determination with Rose Bengal (Müller, 1991).

### Cytotoxicity (MTS)

**Figure 2:**
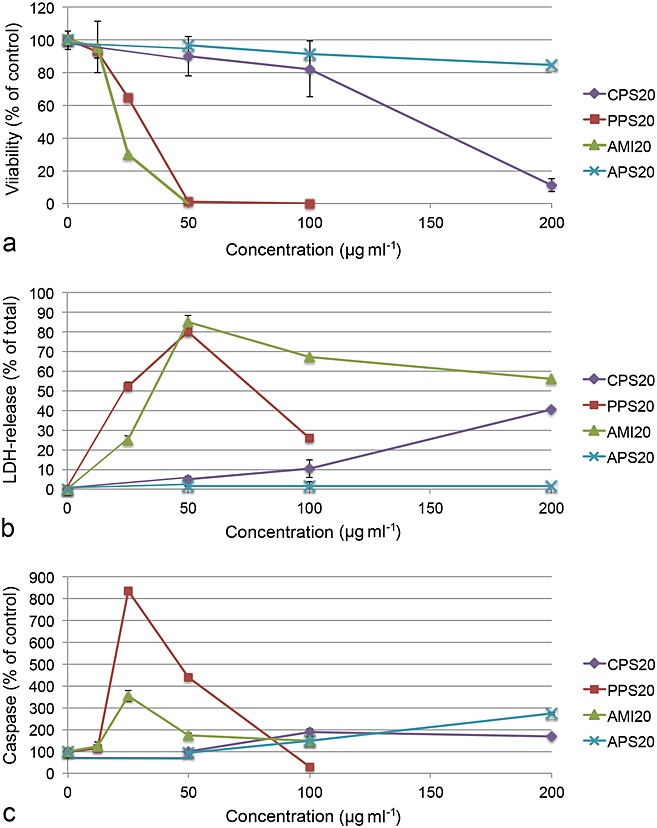
EAhy926 cells exposed to PS particles for 24 h and assessed for viability (MTS, a) and membrane integrity (LDH release, b). Testing for apoptosis by caspase 3/7 activation (c) was performed after 8 h. The most cytotoxic particles PPS20 and AMI20 particles are characterized by decrease in viability and increase of LDH release and caspase 3/7 activation. Means ± SD of four independent experiments are shown. LDH, lactic dehydrogenase.

Cytotoxicity screening with EAhy926 cells was performed to identify toxic ranges of NPs and in SH-SY5Y cells used for the measurements of intracellular [Ca^2+^] levels. Small particles of all charges acted more cytotoxic than 200 nm particles (Fig.2a, Table2). Among the small particles, PPS20 and AMI20 were more cytotoxic than CPS20 and APS20 particles. There was no significant decrease in viability after exposure up to 200 µgml^−1^ PS particles of 200 nm of size (Fig.S, Supplementary Material).

**Table 2:** Overview of lowest particle concentrations at which a significant effect in the exposures for 24 h was noted. Effects up to a concentration of 100 µg ml^−1^ as upper limit for realistic levels are listed

Particles	Viability	Membrane damage		Apoptosis		Oxidative stress	Lysosome damage	Ca^2+^ levels
	MTS EAhy926 (SH-SY5Y)	LDH	PI	Caspase	YoPro	DHE	Lyso-ID	Fura-2
CPS20	(100)	100		100	50			75
PPS20	25 (75)	25	25	25	25	100	10	50
AMI20	25 (50)	25	25	25	25	100		25
APS200							40	
Aero200	50 (50)	50	50	25				500
OX50	(100)	50	50	50	50			125

DHE, dihydroethidium; LDH, lactic dehydrogenase; PI, propidium iodide.

**Figure 3:**
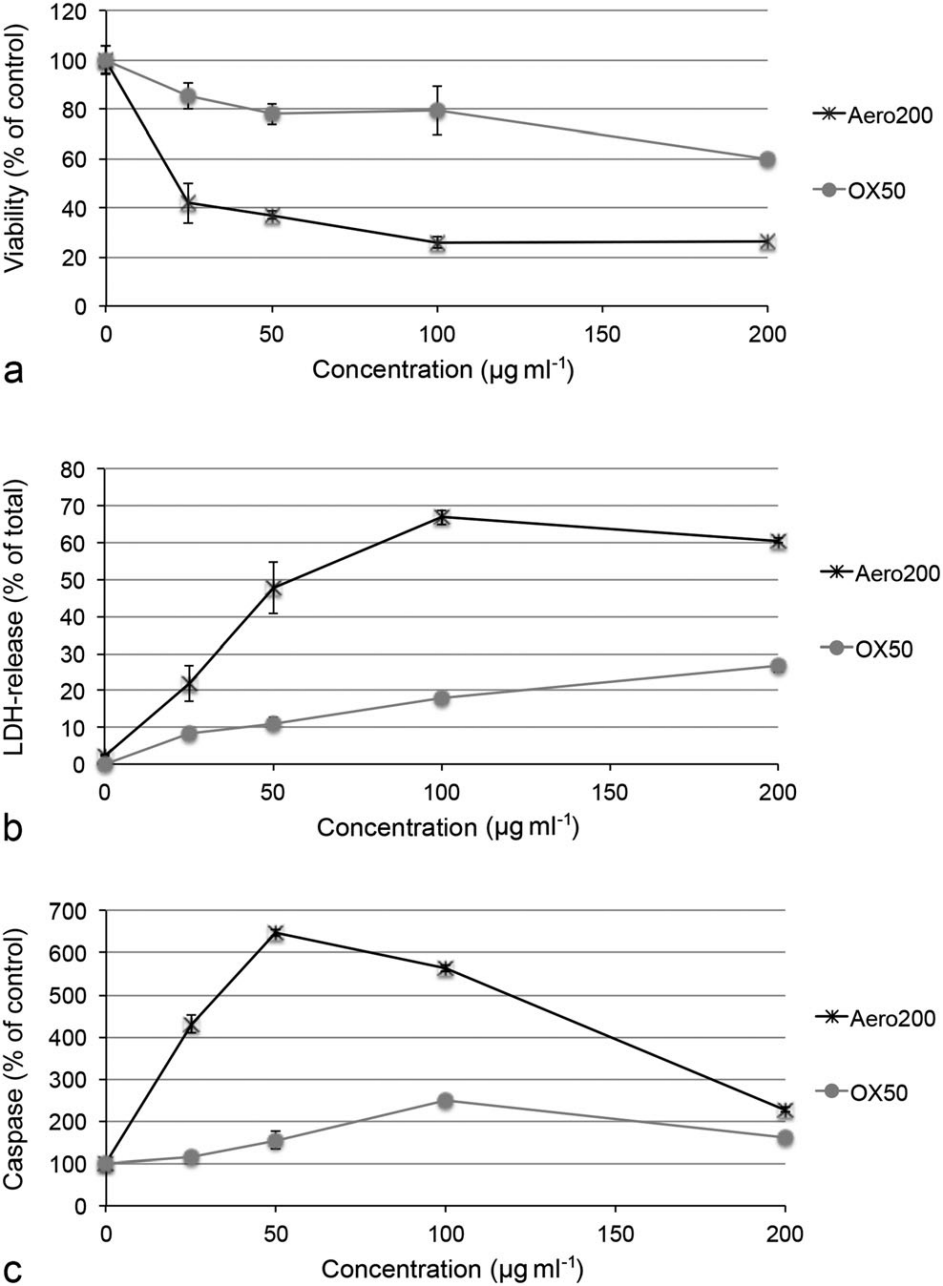
Viability (a), membrane integrity (b) and apoptosis (c) in EAhy926 cells exposed to Aerosil particles. Viability and membrane integrity were assessed by MTS and LDH release after 24 h of exposure and caspase 3/7 activation after 8 h of exposure. Aerosil200 (Aero200) particles are characterized by a steeper decrease in viability and higher increases in LDH release and caspase 3/7 activation. Means ± SD of four independent experiments are shown. LDH, lactic dehydrogenase.

Aero200 particles caused a stronger decrease in EAhy926 cell viability than OX50 particles (Fig.3a, Table2). Sensitivity to all particles was more pronounced in EAhy926 endothelial cells than in SH-SY5Y neuronal cells but the pattern was similar (Fig.2S, Supplementary Material).

### Plasma Membrane Integrity (Lactic Dehydrogenase, Propidium Iodide)

**Figure 4:**
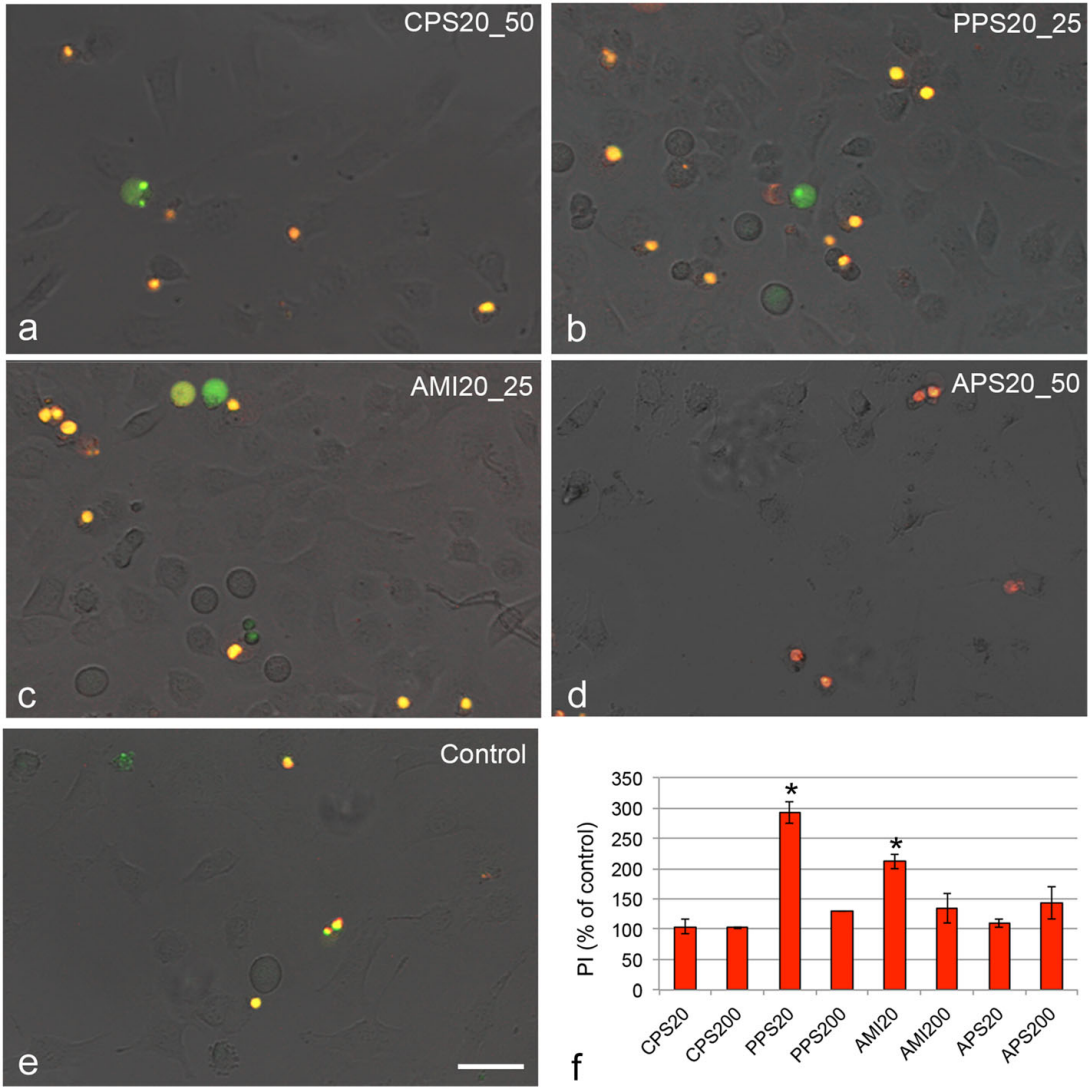
Images of EAhy 926 cells exposed for 24 h to polystyrene particles and stained with YoPro1 (green) as marker for apoptotic cells and PI (red) as marker for necrotic cells. Secondary apoptotic cells show yellow staining. Images were taken at concentrations of the most prominent effects; 25 µg ml^−1^ PPS20 (PPS20_25), AMI20 (AMI20_25) and 50 µg ml^−1^ CPS20 and APS20 particles (CPS20_50; APS20_50) compared to medium as negative control. Quantification of PI staining by fluorometric reading (f) shows staining at the respective concentrations of the 20 nm particles and at 50 µg ml^−1^ of the 200 nm particles. Means ± SD of three independent experiments are shown. Significant changes (*P* < 0.05) compared to the control cells set, as 100%, are marked by asterisk. Scale bar: 20 µm. PI, propidium iodide.

With the exception of the non-cytotoxic APS20 particles, small PS particles caused more membrane damage according to LDH release than larger particles (PPS20>AMI20>CPS20; Fig.2b, Table2). CPS200, PPS200 and AMI200 caused no significant LDH release up to 400 µg ml^−1^, while cells after exposure to 400 µg ml^−1^ APS200 particles released significantly more LDH than cells that were not treated with particles (data not shown). Staining with PI indicated membrane damage of PPS20 and AMI20 particles (Fig.4), while all other particles did not show staining up to 100 µg ml^−1^. The staining with PI was performed in combination with the marker for apoptosis YoPro1 and, therefore, provides some insight into the mechanism. Fifty µg ml^−1^ CPS20 and 25 µg ml^−1^ PPS20 and AMI20 particles showed exclusive YoPro1 staining in some cells. In addition, the negative control shows YoPro1-positive and YoPro1/PI-stained cells. In APS20-exposed cells, mainly PI-stained cells are seen (Fig.4).

Both Aerosil® particles, OX50 and Aero200, showed significant membrane damage according to LDH release (Fig.3b, Table2) and PI staining (data not shown).

### Apoptosis (Caspase 3/7 Activation, YoPro1)

All 20 nm PS particles, except APS20 particles, caused caspase 3/7 activation (Fig.2c). Caspase 3/7 activation was more strongly induced by AMI20 and PPS20 than by CPS20 particles. CPS200, PPS200 and AMI200 induced some caspase 3/7 activation at 400 µg ml^−1^ but no effect was seen in the YoPro1 staining (data not shown).

YoPro1 staining was significantly increased in cells exposed to CPS20, PPS20 and AMI20 particles (Fig.3S, Supplementary Material, Table2). All other PS particles did not show significant increases in the concentration range tested (up to 100 µg ml^−1^).

OX50 and Aero200 particles showed caspase 3/7 activation (Fig.3c), while only OX50 particles showed increased YoPro1 staining (data not shown).

### Lysosomal Damage (Acridine Orange, Lyso-ID)

Changes in lysosome function were studied for particles in non-cytotoxic concentrations by fluorometric reading of the acridine orange staining and by analysis of confocal images of the stained cells. Analysis of confocal images did not reveal significant differences between cells exposed to particles compared to cells not exposed to particles. The ratio of green (high pH)/red (low pH) was only significantly increased in cells exposed to 10 µM chloroquine (positive control). Similarly small, non-significant changes in cells exposed to particles were also seen in the fluorometric analysis (Fig.4S, Supplementary Material). One problem was the high variation between experiments. To verify, whether lack of clear signals might be caused by interference of the particles with the dye, another functional staining for lysosomes was used.

**Figure 5:**
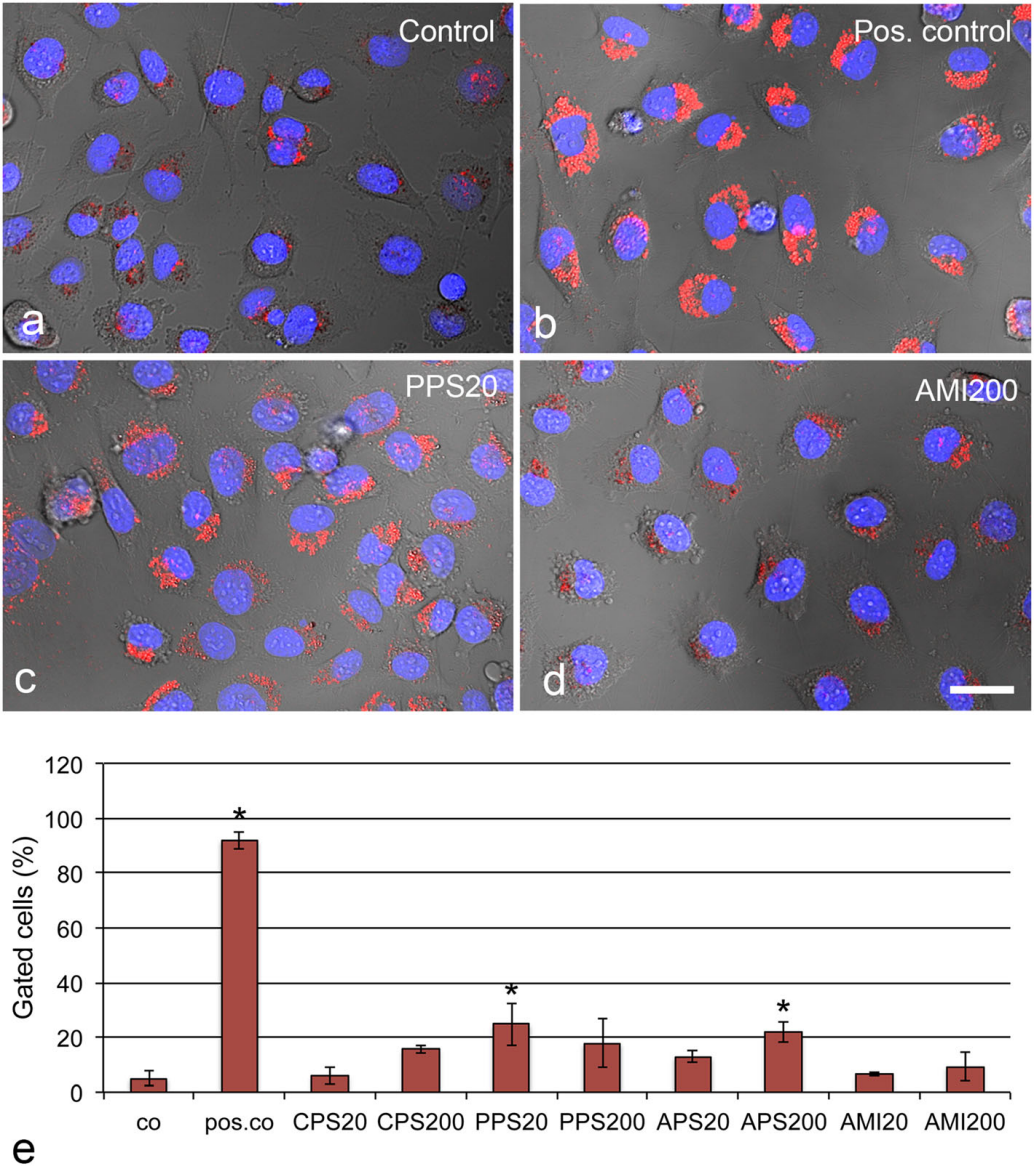
Identification of lysosomal damage caused by 10 µg ml^−1^ polystyrene particles after 24 h, assessed by LysoD (red) staining. Different levels of lysosomal damage are seen in PPS20 (high staining, c) and AMI200 (lower staining, d) particles. Chloroquine serves as positive control (pos. co). Quantification of cellular staining by TissueFAXS software (e) shows lysosomal damage mainly by exposure to PPS20 and AMI200 particles. Means ± SD of three independent experiments are shown. Significant changes (*P* < 0.05) compared to control cells (co) are indicated by asterisk. Scale bar: 20 µm.

Staining with LysoID, the commercial dye for indication of lysosome damage, showed significant increases for PPS20 (Fig.5) and APS200 particles (Table2). All other PS particles did not show significant increases in the concentration range tested (up to 40 µg ml^−1^).

Aero200 and OX50 particles did not cause any significant changes in both lysosomal function assays up to a concentration of 100 µg ml^−1^ (data not shown).

### Oxidative Stress (Dihydroethidium)

Oxidized DHE integrated in the nuclei of oxidatively damaged cells was determined up to concentrations of 100 µg ml^−1^ for the cytotoxic AMI20, CPS20 and PPS20 particles and up to 300 µg ml^−1^ of the other PS particles. PPS20, AMI20 and APS200 particles showed significant increases in the fluorescent signal (Fig.5S, Table2).

OX50 and Aero200 particles did not cause increases in DHE fluorescence up to a concentration of 100 µg ml^−1^ (data not shown).

### Intracellular [Ca^2+^] Levels (Fura-2)

SH-SY5Y is a subcloned cell line derived from the SK-N-SH neuroblastoma cells. These cells are popular models for neurodegenerative disorders as they can be differentiated to various types of functional neurons by the addition of specific compounds (Kovalevich & Langford, 2013). In this study, their ability to increase intracellular [Ca^2+^] levels as a response to toxicants was exploited for the screening. For neurotoxicity studies, non-differentiated SH-SY5Y cells were found to be more suitable because they showed a higher sensitivity to toxicants than the differentiated cells (Cheung *et al*., 2009).

**Figure 6:**
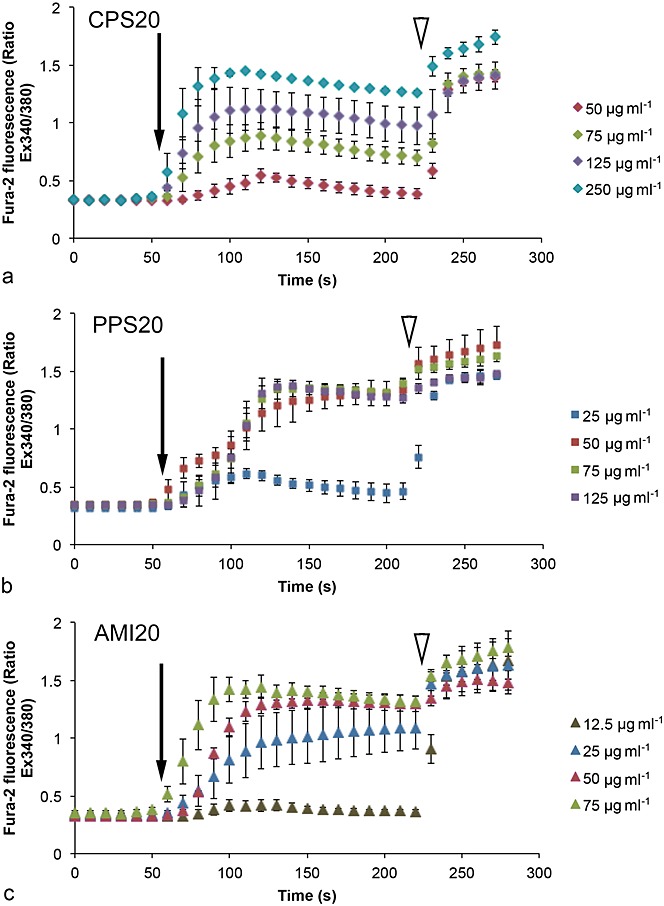
Measurements of Ca^2+^ levels in SH-SY5Y cells upon exposure to polystyrene particles with the use of fura-2. Changes in fura-2 fluorescence were expressed as ratio of emission intensities at the two excitation wavelengths (340 vs. 380 nm). Results are means ± SD of three independent experiments with 40–50 cells per recording. For statistical analysis, data at 120, 150 and 180 s were used. The arrow indicates start of perfusion with NPs, the arrowhead start of perfusion with ionomycin for depletion of Ca^2+^ stores.

Superfusion with HBSS alone did not cause any increases of intracellular [Ca^2+^] levels showing absence of mechano-activation of ion channels. Significant increases of intracellular [Ca^2+^] levels were seen in cells exposed to CPS20, PPS20 and AMI20 (Fig.6, Table2). Increases were seen at lower concentrations for PPS20 and AMI20 particles than for CPS20 particles. Two hundred nm sized particles did not increase intracellular [Ca^2+^] levels (data not shown). APS particles showed such a high degree of aggregation that evaluation was not possible.

Increases of intracellular [Ca^2+^] levels were observed at lower particles concentrations of OX50 than of Aero200 particles. The increases were significant for incubations with 125 µg ml^−1^ OX50 and 500 µg ml^−1^ Aero200 particles (Fig.6S, Supplementary Material).

## Discussion

Twenty nm unmodified, carboxyl and amine functionalized PS particles as well as 12 nm and 40 nm fumed silica particles caused a decrease in viability, apoptosis and disruption of membrane integrity. Two hundred nm PS particles, except amidine functionalized particles did not cause any adverse cellular effects up to a concentration of 200 µg ml^−1^. Measurement of intracellular [Ca^2+^] levels identified cytotoxic particles and could differentiate between cytotoxic particles causing non-cytotoxic membrane damage and severe membrane disruption. There was no clear link between increased intracellular [Ca^2+^] levels and lysosomal damage or ROS generation.

This study aimed to elucidate the role of particle properties, namely size, surface charge and hydrophilicity, on cytotoxicity. Several studies report higher cytotoxicity of positively charged NPs but comparison of differently charged particles is complicated by the fact that charge density, stability in suspension and other surface properties also play a role (Fröhlich, 2012). In this study, positive and neutral AMI20 and PPS20 particles reacted similarly in the assays after 24 h exposure and regarding increases in intracellular [Ca^2+^] levels. The more positively charged APS20 particles did not cause any adverse cellular effects up to 100 µg ml^−1^. This was due to much stronger agglomerate formation of these particles compared to the other 20 nm PS particles in all media. Stability of the NP suspension and tendency for agglomeration may differ (Meyer-Plath & Schweinberger, 2014). Agglomerate formation has been shown to increase cytotoxic and genotoxic effects of titanium dioxide particles (Magdolenova *et al*., 2012), while there was a decreased cytotoxicity and inflammatory response in studies on boehmite NPs (Forest *et al*., 2014). Increased cytotoxicity of alumina NP aggregates has been reported in adherent cells not in cells growing in suspension, supporting the hypothesis that agglomerates with a greater tendency for sedimentation increase the exposure dose for the adherently growing cells (Yoon *et al*., 2011). As these effects are also dynamic in nature, it is difficult to identify the effects of different media, the cell types and degree of sedimentation on the obtained results. Therefore, all statements on the impact of size and surface charge in this study are limited by these interactions. Experiments in this study used protein-free media to decrease protein-induced agglomeration of unmodified, positively charged and negatively charged PS particles (Casado *et al*., 2013; Fröhlich *et al*., 2012; Guarnieri *et al*., 2011).

As expected, small particles in this study caused more adverse cellular effects than the larger particles. This finding is in line with the majority of reports comparing smaller and larger particles and is mostly explained by the higher reactive surface and, in case of soluble metal particle, greater release of metal ions, of the smaller particles (for more detail see reviews, e.g., Fröhlich, 2013; Podila & Brown, 2013). The overview in Table2 shows that the small CPS20, PPS20 and AMI20 particles caused significant effects in more assays. These particles, however, were not always reactive in all assays. Lysosome damage was induced only by PPS20 particles and not by AMI20 particles. As both size and hydrophilicity were similar for these particles, different binding of cytoplasmic proteins appears a likely reason for the observed action. It is known that proteins bind to neutral particles in lower degree than to (preferentially negatively) charged particles (Deng *et al*., 2013; Lee & Cheng, 2013). Owing to the lower extent of protein binding PPS20 particles might be more reactive than AMI20 particles. Another potential reason might be a different delivery of the particles to lysosomes. While clathrin-mediated uptake and macropinocytosis deliver the content to lysosomes, particles, which were ingested caveolin-mediated can be delivered either to lysosomes or to the endoplasmic reticulum (Fröhlich & Roblegg, 2012). Inhibition studies demonstrated predominant uptake of positively charged NPs via clathrin and macropinocytosis, while negatively charged NPs were mainly ingested by caveolin-mediated uptake (Bannunah *et al*., 2014). The preference of positively charged NPs for clathrin-mediated uptake and of negatively charged for caveolin-mediated uptake was confirmed by other studies (Bhattacharjee *et al*., 2013). Therefore, the higher lysosome damage by unmodified PPS20 particles cannot be explained by preferential clathrin-mediated uptake. On the other hand, higher total cellular uptake of PPS20 than of AMI20 particles cannot be excluded. Direct comparison is not possible because fluorescently labeled AMI20 particles were not available from the supplier. In contrast to studies that reported that (preferentially positively) charged NPs were taken up to higher degree than neutral NPs (Jiang *et al*., 2011), our comparison between fluorescently labeled CPS20 and PPS20 particles showed that after 24 h of exposure EAhy926 cells ingested 4.7 times more PPS20 particles than CPS20 particles (unpublished data). The higher cellular uptake and/or higher intracellular reactivity are supported by the significantly higher ROS production by PPS20 than by AMI20 particles (Fig.5S).

Amidine functionalized PS particles were the only 200 nm PS particles that impaired lysosome function. This behavior might be explained by a higher positive surface charge of the APS200 particles demonstrated by the highly positive zeta potential in water (Table1). The positive surface charge of particles has been correlated to increased cellular uptake and higher biological activity (Swami *et al*., 2012). It is also known that positively charged particles can increase the intralysosomal pH causing impairment of their function and even rupture of these organelles (Nel *et al*., 2009). NPs developed for intracellular delivery exploit this mechanism, which has been termed “endosomal escape,” to prevent degradation of the delivered molecules in lysosomes (Panyam & Labhasetwara, 2003). It is possible that the APS200 particles tested in this study displayed their adverse effect on lysosomes through this mechanism.

The most common mode of cytotoxic action of non-biodegradable NPs is generation of ROS (Manke *et al*., 2013). Increased oxidation of DHE was only observed at cytotoxic concentrations of plain (PPS20) particles and amine functionalized (AMI20) particles. Other studies also did not find indications for intracellular ROS production by PS particles and by Aero200 and OX50 particles in non-phagocytic cells (Fröhlich *et al*., 2009; Gehrke *et al*., 2013; Xia *et al*., 2008).

Measurement of intracellular [Ca^2+^] levels is not a common parameter in cytotoxicity testing of NPs but might provide additional information on membrane effects of NPs after short contact times. These contact times are typical for intravenous application. It is important to know whether routine cytotoxicity testing identifies the same particles as cytotoxic as the fast contact screening. SH-SY5Y neuroblastoma cells were used in the screening based on their higher sensitivity than EAhy926 and A549 cells (data no shown). These cells have been often used in neurotoxicity research (Cheung *et al*., 2009). In this study we used the traditional imaging protocol, where salt solutions, mostly HBSS, is used (Bootman *et al*., 2014). Particle concentrations, at which a cellular action was seen, cannot be directly compared to concentrations used in other assays because the contact time between plasma membrane and particles is much shorter than in conventional screening assays (30 s compared to 24 h). Despite these differences, measurement of intracellular [Ca^2+^] levels was able to differentiate between particles with moderate and severe membrane damage. The most likely reason for the observed increases in intracellular [Ca^2+^] levels is the induction of membrane repair (Sharei *et al*., 2014). Two facts may explain the absence of such increases after severe membrane damage by NPs. One reason is that no repair mechanisms are initiated because the damage is too severe. The other cause may be that the large holes in the plasma membrane allow leakage of the FURA-2 dye. Both mechanisms can explain why the more cytotoxic Aerosil ®200 particles did not induce increases in intracellular [Ca^2+^], while the less cytotoxic Aerosil ®OX50 particles caused such a reaction. Increases in intracellular [Ca^2+^] levels correlated well with YoPro1 staining. YoPro1 is an early marker for membrane changes in apoptosis but also identifies distortion of the lipid layer, independent of apoptosis (Wlodkowic *et al*., 2011).

## Conclusions

For the majority of particles 24 h cytotoxicity and measurements of intracellular [Ca^2+^] levels identified the same particles as cytotoxic. Cytotoxicity of PS particles was more determined by size than by surface charge. Only for the 200 nm PS particles, a positive surface charge was correlated to adverse cell effects. Measurements of intracellular [Ca^2+^] levels might give additional information on the interaction of NPs with membranes.

## Supporting Information

Supporting info item
