# Supplementary material for: Intracellular calcium levels as screening tool for nanoparticle toxicity
Source: J Appl Toxicol. 2015 May 14;35(10):1150–9. doi: 10.1002/jat.3160 (PMC4606983; doi:10.1002/jat.3160)
Supplement: Supplementary file 1 [file jat0035-1150-sd1.doc]

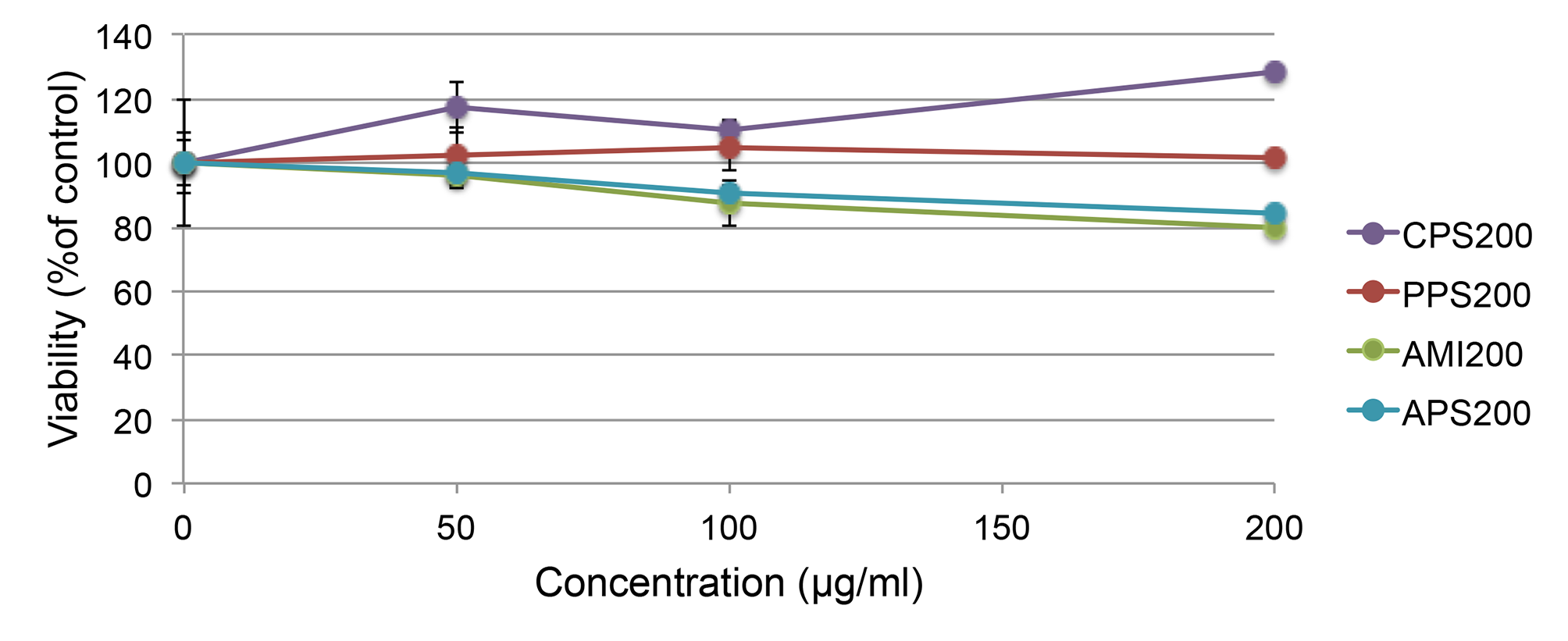


Fig. 1s: Viability of EAhy926 cells detected by MTS assay after exposure for 24h to 200 nm polystyrene particles


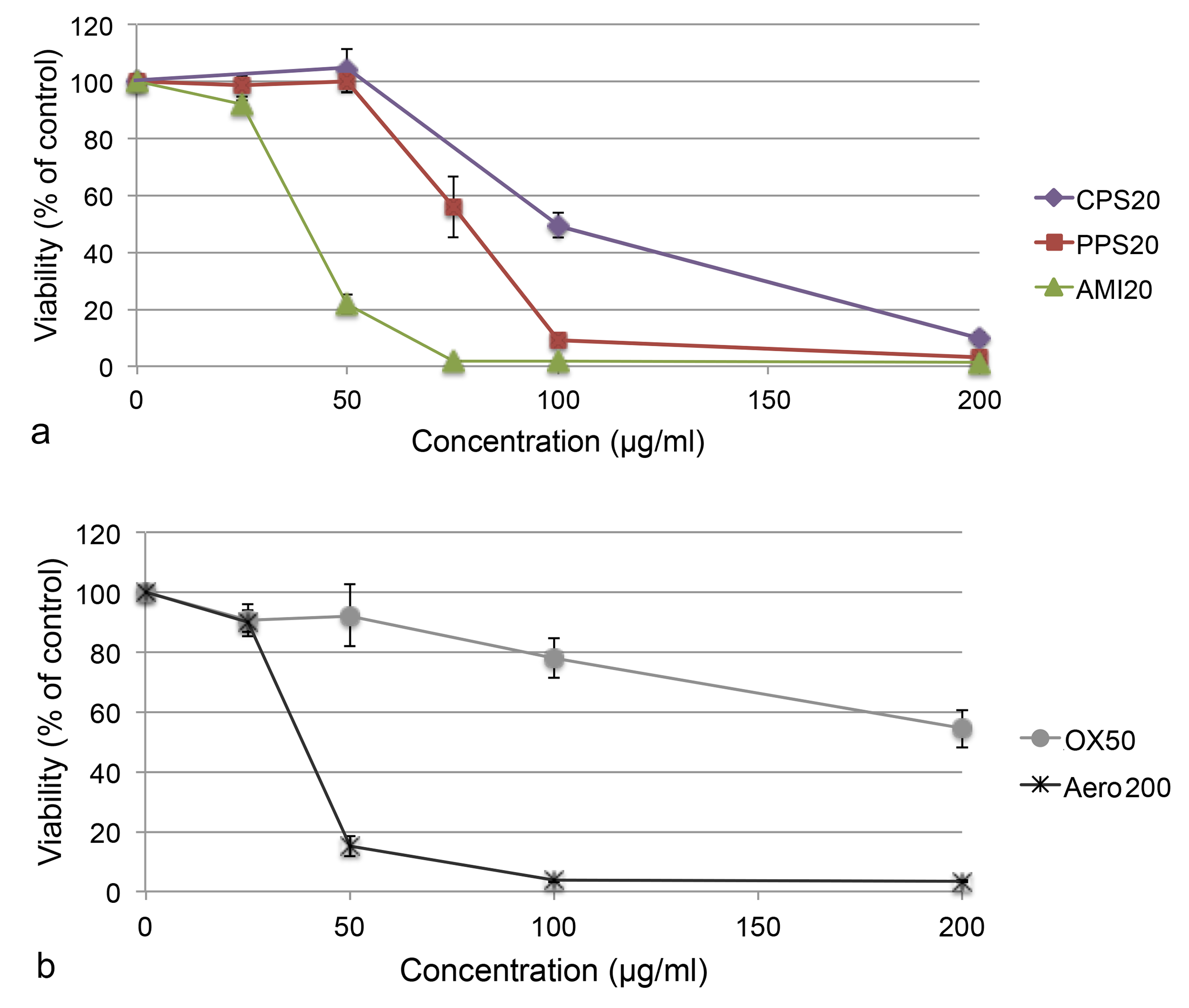


Fig. 2s: Viability of SH-SY5Ycells detected by MTS assay after exposure for 24h to polystyrene (a) and silica particles (b). Viability is normalized to untreated cells as 100%.


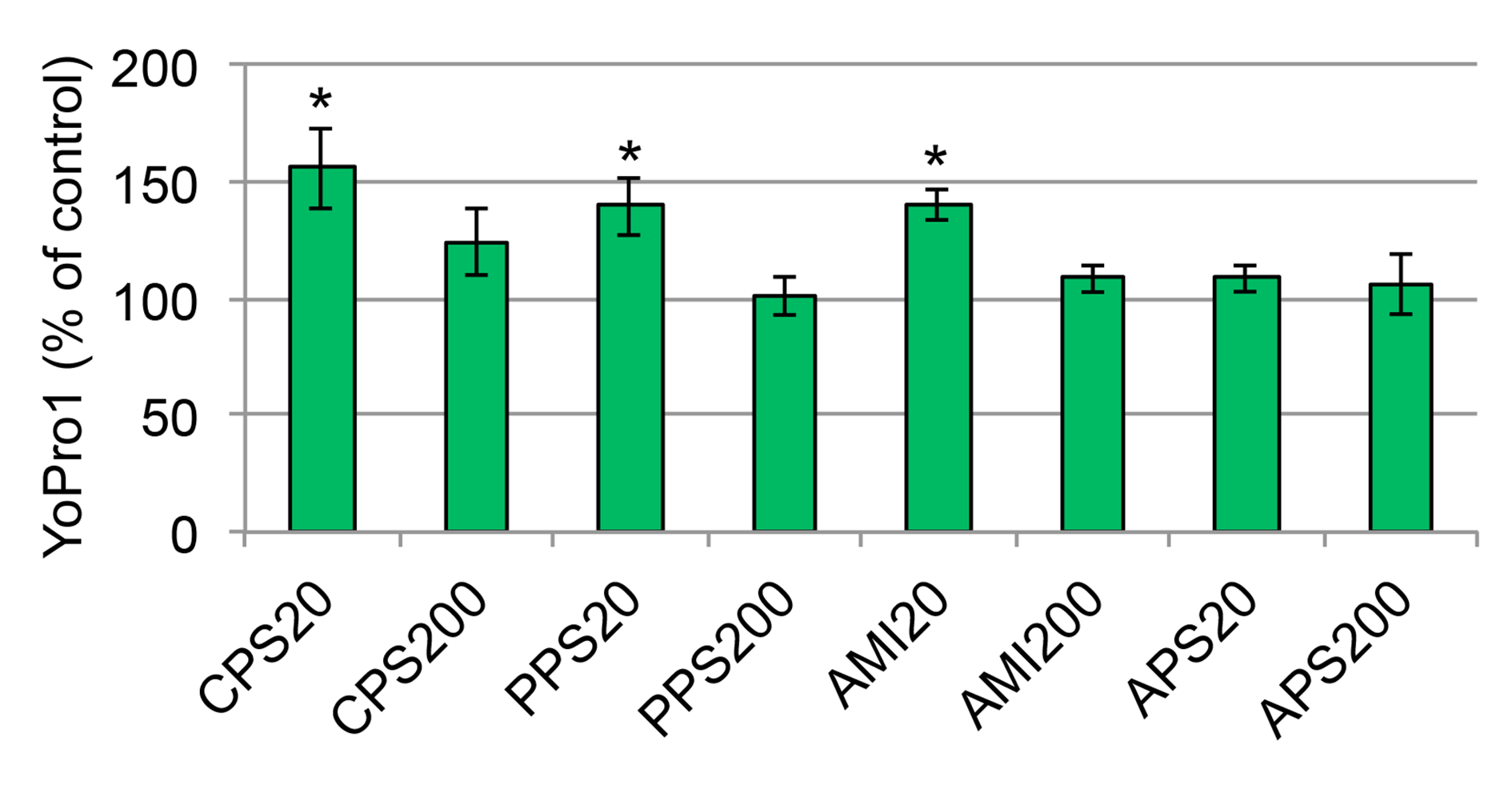


Fig. 3s: Fluorometric quantification of YoPro1 staining in EAhy926 cells exposed for 24h to PS particles in the most effective concentrations (12.5 µg/ml PPS20, 25 µg/ml AMI20, 50 µg/ml CPS20, APS20, CPS200, PPS200, AMI200, and APS200). Significant changes compared to the control (no particles) are indicated by asterisk.


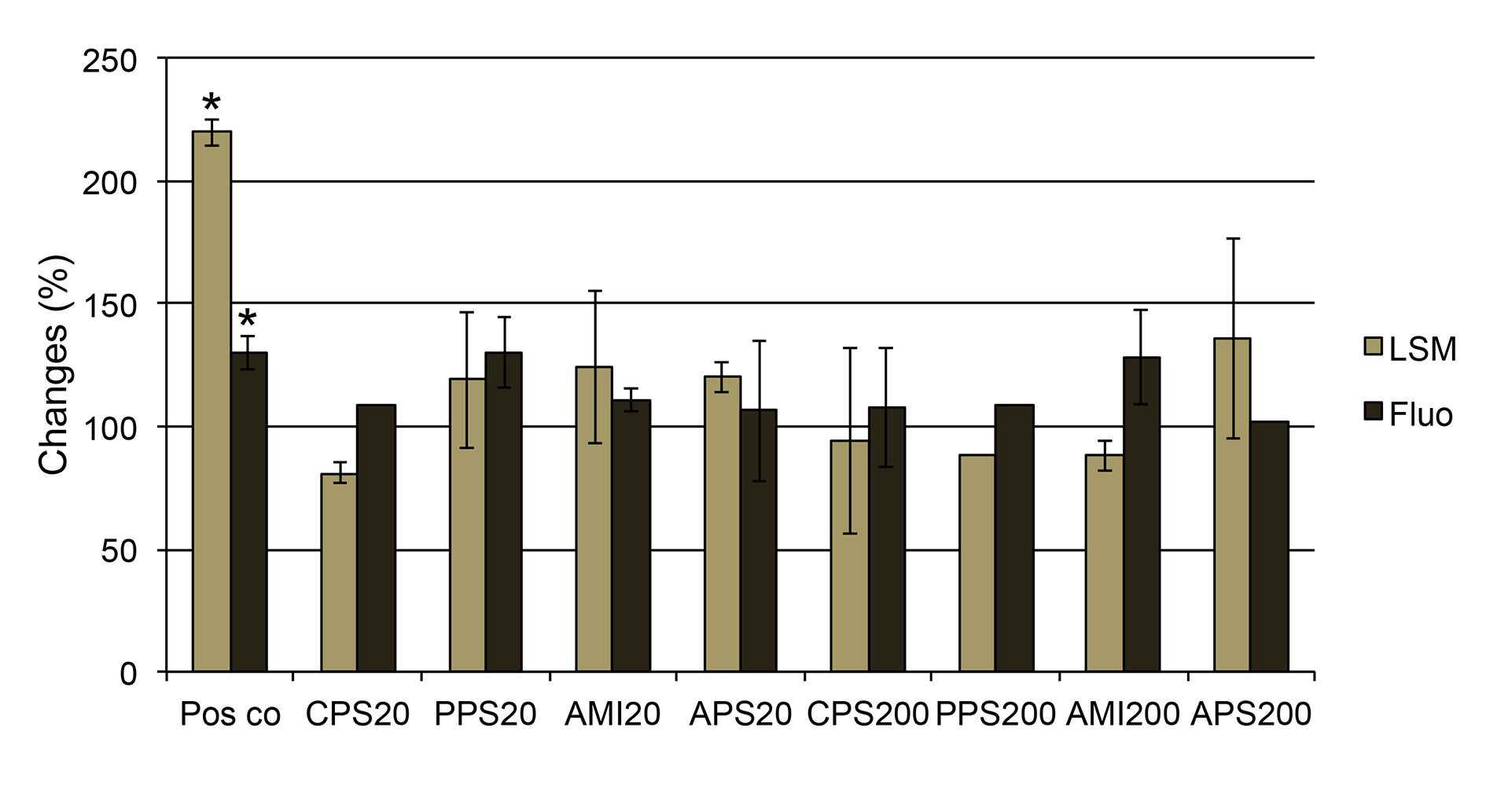


Fig. 4s: Comparison of analysis of lysosomal changes in EAhy926 cell stained with Acridine Orange after 24h of exposure to 10 µg/ml polystyrene particles. Analysis by fluorometric reading (Fluo) and by analysis of confocal images with Image J (LSM). Positive control (pos co) is chloroquine. Significant changes are marked by asterisk.


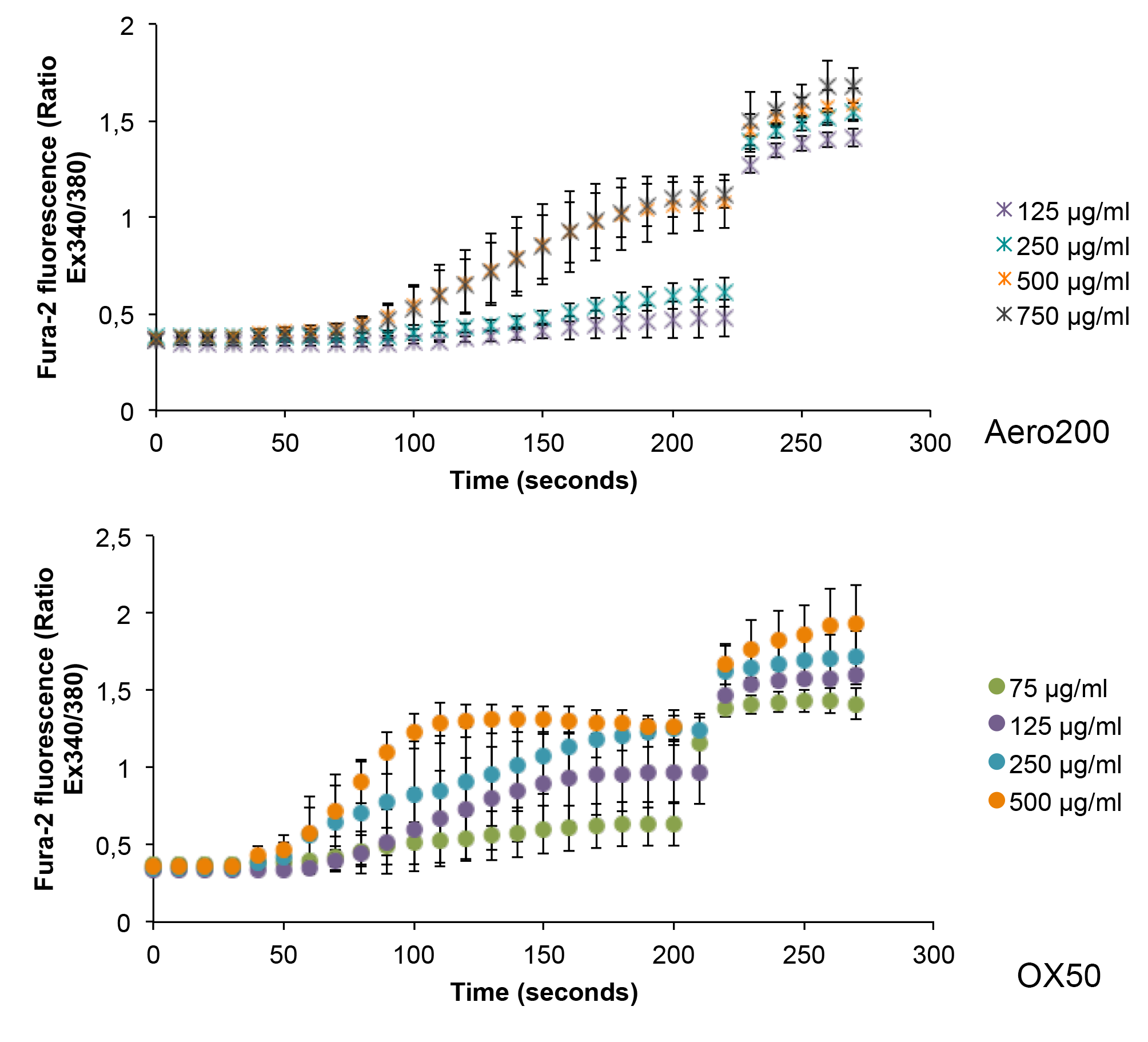


Fig. 5s: Changes in intracellular [Ca^2+^] levels in SH-SY5Ycells exposed to different concentrations of Aerosil200 (Aero200) and Aerosil OX50 particles according to ratio imaging with Fura-2. Results are means ±SD of 3 independent experiments with 40-50 cells per recording. Changes compared to untreated controls were analyzed for significance at 120, 150, and 180 sec of incubation with particles.
